# Supplementary material for: Distinct mechanisms of neutralization by antibodies targeting a conserved pneumovirus F epitope
Source: bioRxiv. 2026 Sep 24:2026.09.21.749132. Preprint. [Version 1] doi: 10.64898/2026.09.21.749132 (PMC13622162; doi:10.64898/2026.09.21.749132)
Supplement: Supplementary file 1 [file media-1.pdf]

Supplementary Information

Distinct mechanisms of neutralization by antibodies targeting a conserved pneumovirus F epitope

Sebastian Ols, Rodrigo Arcoverde Cerveira, Andrew J. Borst, Erick Bermúdez-Méndez, Florian Gegenfurtner, Elise Eray, Connor Weidle, Marcos C. Miranda, Zhitong Peng, Kenneth Carr, Rebecca Skotheim, Jana Kochmann, Natalie Brunette, Klara Lenart, Leo Hanke, Gunilla B. Karlsson Hedestam, Laurent Perez, Aleksandar Antanasijevic, Neil P. King, and Karin Loré

*Lead contact: sebastian.ols@ki.se

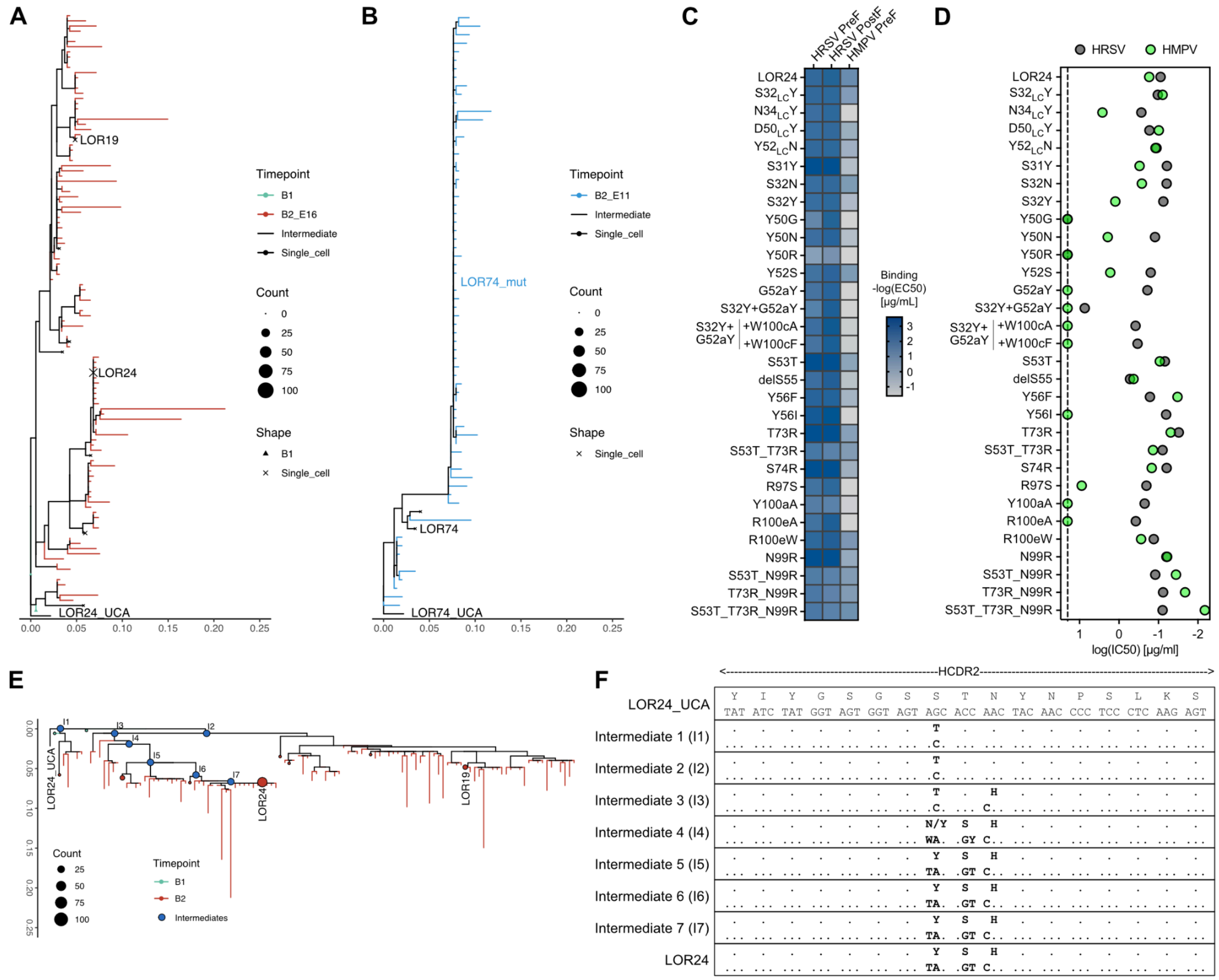

Figure S1. LOR24 lineage development and affinity maturation for acquisition of potency and breadth. Related to Figure 1, 2, and 6.

(A) Maximum-likelihood phylogenetic tree of LOR24 and LOR19 lineage development, rooted on the LOR24_UCA.

(B) Maximum-likelihood phylogenetic tree of LOR74 and LOR74_mut lineage development, rooted on the LOR74_UCA.

(C) Binding of recombinantly expressed LOR24 variants by ELISA assayed against HRSV preF, HRSV postF and HMPV preF. Binding is visualized as a heatmap of -log(EC50) values derived from fitted binding curves.

(D) HRSV and HMPV neutralization log(IC50) values for recombinantly expressed LOR24 variants.

(E) Maximum-likelihood phylogenetic tree of LOR24 and LOR19 lineage development, rooted on the LOR24_UCA. Intermediates in the evolution of LOR24 from the UCA to its mature sequence are indicated.

(F) Nucleotide and amino acid sequence alignment of LOR24's HCDR2 evolution with LOR24_UCA as reference. Dots indicate identical nucleotides and amino acid residues.

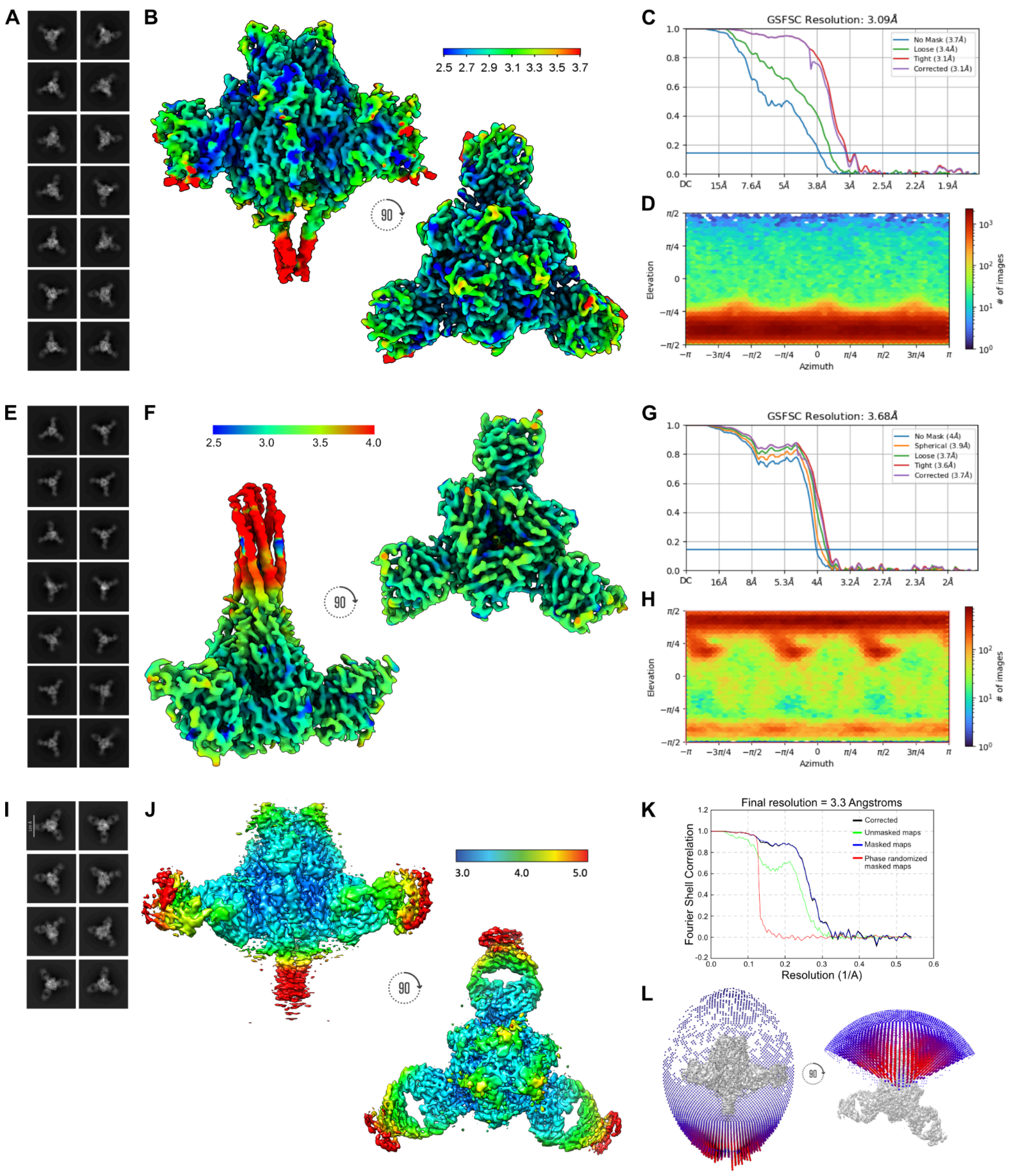

Figure S2. CryoEM structure determination of LOR24 and LOR69 in complex with pneumovirus F antigens. Related to Figures 2, 5, and 6.

(A-D) LOR24 Fab in complex with HRSV preF.

(E-H) LOR24 Fab in complex with HRSV postF.

(I-L) LOR69 Fab in complex with HRSV preF.

(A, E and I) Representative 2D class averages.

(B, F and J) Local resolution maps.

(C, G and K) Global gold-standard Fourier shell correlation (GSFSC) plots.

(D, H and L) Orientational distribution plots.

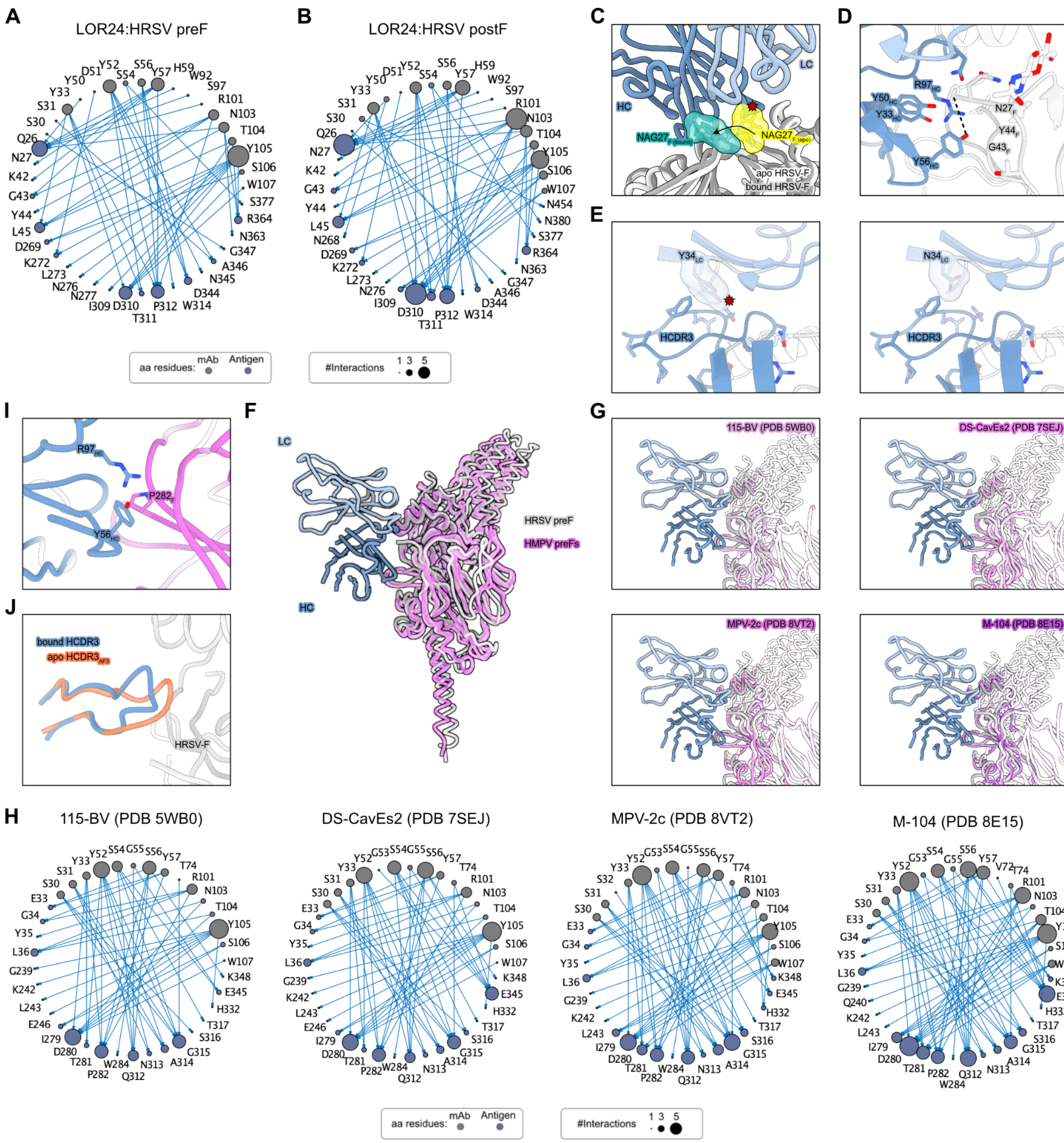
A
LOR24:HRSV preF
B
LOR24:HRSV postF
C
LC
HC
NAG27
apo HRSV-F
bound HRSV-F
D
R97
Y50
Y33
Y56
N27
Y44
G43
E
Y34
N34
HCDR3
aa residues: mAb Antigen
#Interactions 1 3 5
I
R97
P282
Y56
F
LC
HC
HRSV preF
HMPV preFs
G
115-BV (PDB 5WB0)
DS-CavEs2 (PDB 7SEJ)
MPV-2c (PDB 8VT2)
M-104 (PDB 8E15)
J
bound HCDR3
apo HCDR3
HRSV-F
H
115-BV (PDB 5WB0)
DS-CavEs2 (PDB 7SEJ)
MPV-2c (PDB 8VT2)
M-104 (PDB 8E15)
aa residues: mAb Antigen
#Interactions 1 3 5

Figure S3. Structural basis for LOR24 binding to HRSV-F and HMPV-F. Related to Figures 2 and 3.
(A and B) Network map of LOR24:HRSV preF (A) and LOR24:HRSV postF (B) interactions as determined by the Epitope Analyzer tool.[1]
(C) Overlay of apo postfusion HRSV-F (PDB 3RRR) on the LOR24-bound postfusion HRSV-F cryoEM structure showing that the N27 glycan (yellow) would clash with LOR24 and that it is repositioned in the bound structure (seagreen).
(D) Magnified view of $Y56_{HC}$-$R97_{HC}$ cation-π interaction and π-stacking network of $Y56_{HC}$ with $Y33_{HC}$ and $Y50_{HC}$.
(E) $Y34_{LC}N$ substitution alleviates strains in backbone packing of the HCDR3.
(F and G) Overlay of LOR24-bound prefusion HRSV-F with different prefusion HMPV-Fs.
(H) Network map of LOR24 interactions with different prefusion HMPV-Fs as determined by the Epitope Analyzer tool.[1]
(I) Magnified view of $Y56_{HC}$-$R97_{HC}$ interactions with prefusion HMPV-F (MPV-2c, PDB 8VT2).
(J) HCDR3 of LOR24 folding back onto itself compared to the extended conformation of the apo structure predicted by AlphaFold3.[2]

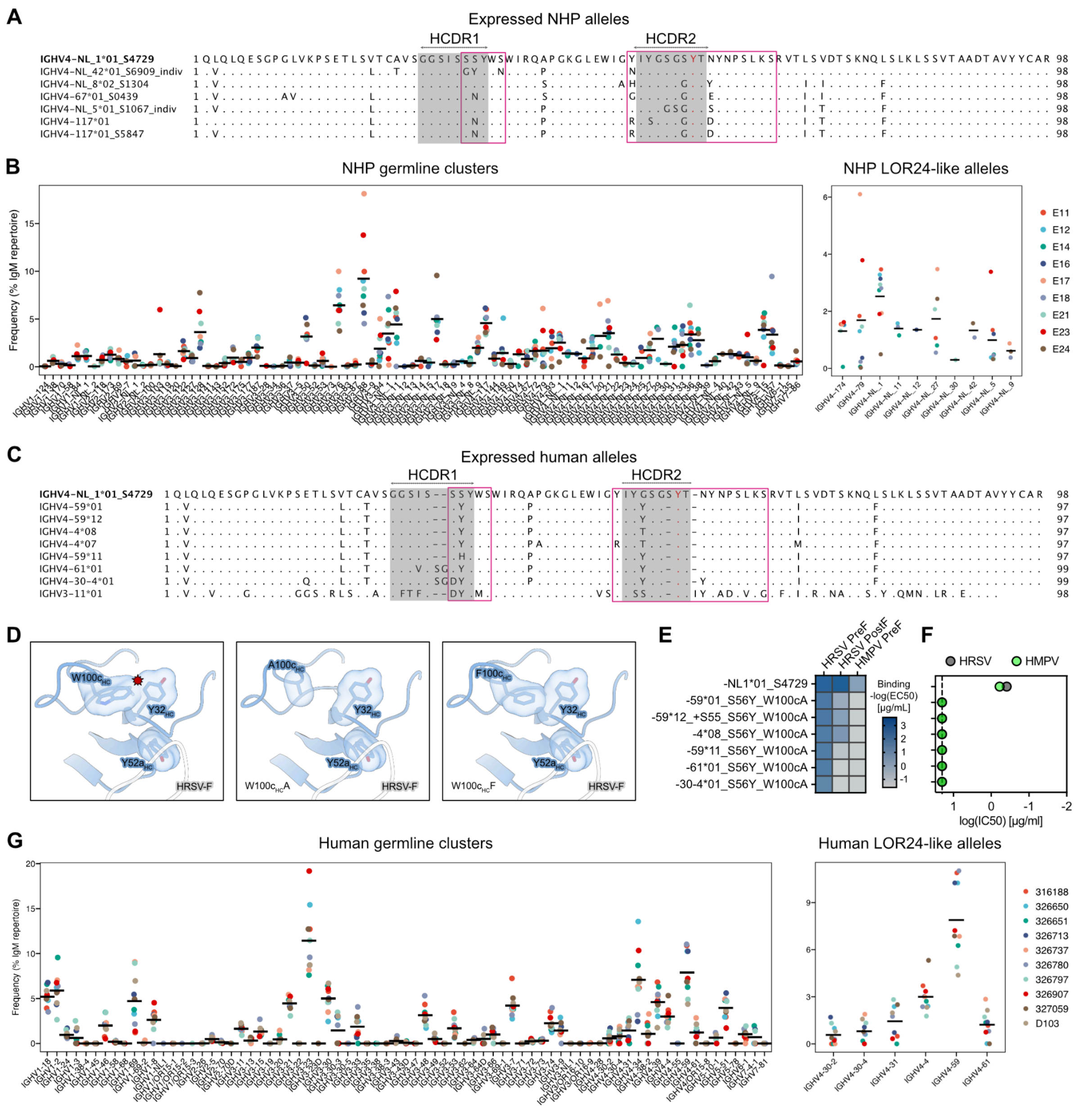

Figure S4. NHP and human IGHV alleles may accommodate an LOR24-like response. Related to Figure 4.

(A and C) Amino acid sequence alignment of IGHV NHP (A) and human (C) alleles selected and tested experimentally based on high similarity to key residues of LOR24. LOR24 germline is set as the reference. Boxes indicate the CDRs according to IMGT (gray) and Kabat (pink).

(B and G) IgM allele frequencies and cumulative LOR24-like frequencies from the repertoires of nine NHPs[3] (B) and ten humans[4] (G).

(D) Magnified view of $S32_{HC}Y$ clash with $W100c_{HC}$ induced by $G52a_{HC}Y$. Substituting $W100c_{HC}$ for less bulky hydrophobic residues ($W100c_{HC}A$ and $W100c_{HC}F$) may resolve this clash.

(E) Binding of recombinantly expressed W100cA human variants by ELISA assayed against HRSV preF, HRSV postF and HMPV preF. Binding is visualized as a heatmap of -log(EC50) values derived from fitted binding curves.

(F) HRSV and HMPV neutralization log(IC50) values for recombinantly expressed W100cA human variants.

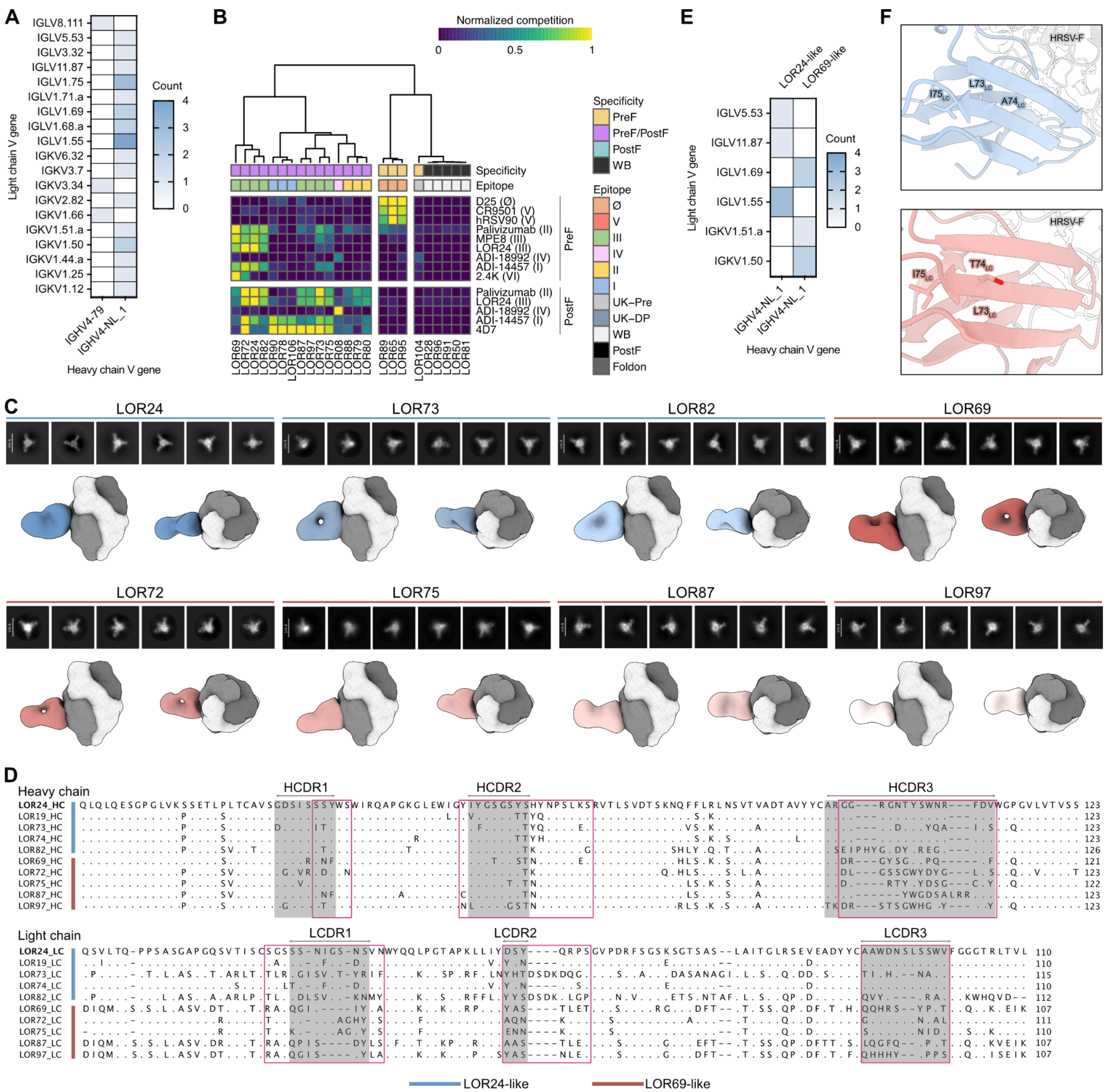

Figure S5. LOR24 and LOR69 define two distinct antibody classes elicited by immunization. Related to Figure 5.

(A) Heatmap of heavy and light chain V gene pairing for expressed LOR24-like mAbs.

(B) Heatmap of epitope binning by competition ELISA assayed on HRSV preF and HRSV postF against a panel of reference mAbs. WB, weak binder; UK, unknown; DP, double positive.

(C) Representative 2D class averages and nsEM 3D reconstructions of site III NHP mAbs. mAbs could be classified by their orthogonal binding mode as either LOR24-like or LOR69-like.

(D) Amino acid sequence alignment of LOR24-like and LOR69-like mAbs with LOR24 set as the reference. Boxes indicate the CDRs according to IMGT (gray) and Kabat (pink).

(E) Heatmap of heavy and light chain V gene pairing for LOR24-like and LOR69-like mAbs.

(F) Magnified views of the 3-mer motif in LFR3 that discriminates LOR24-like from LOR69-like mAbs.

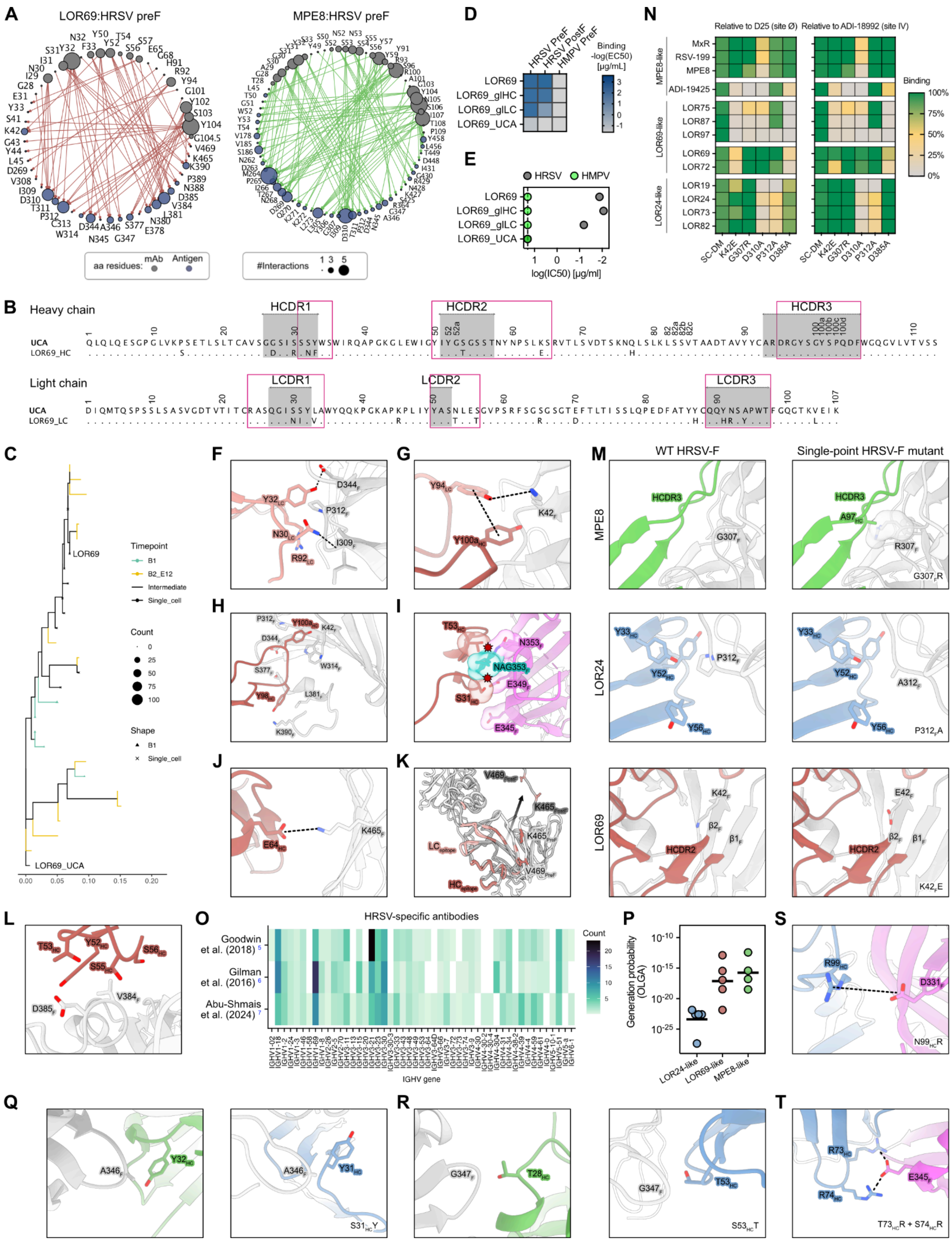

A
LOR69:HRSV preF
MPE8:HRSV preF
aa residues:
mAb
Antigen
#Interactions
1 3 5
B
Heavy chain
HCDR1
HCDR2
HCDR3
UCA
LOR69_HC
QLQLQESGPGLVKPSETLSLTCAVSGGSISSSYWSWIRQAPGKGLEWIGYIYGSGSSTNYNPSLKSRVTLSVDTSKNQLSLKLSSVTAADTAVYYCARDRGYSGYSPQDFWGQGVLVTVSS
Light chain
LCDR1
LCDR2
LCDR3
UCA
LOR69_LC
DIQMTQSPSSLSASVGDTVTITCRASQGISSYLAWYQQKPGKAPKPLIYYASNLESGVPSRFSGSGSGTEFTLTISSLQPEDFATYYCQQYNSAPWTFGQGTKVEIK
C
LOR69
LOR69_UCA
Timepoint
B1
B2_E12
Intermediate
Single_cell
Count
0
25
50
75
100
Shape
B1
Single_cell
D
HRSV PreF
HRSV PostF
HMPV PreF
Binding -log(EC50) [μg/mL]
LOR69
LOR69_glHC
LOR69_glLC
LOR69_UCA
E
HRSV
HMPV
log(IC50) [μg/ml]
F
G
H
I
J
K
L
M
WT HRSV-F
Single-point HRSV-F mutant
MPE8
LOR24
LOR69
G307$_F$R
P312$_F$A
K42$_F$E
N
Relative to D25 (site Ø)
Relative to ADI-18992 (site IV)
MPE8-like
LOR69-like
LOR24-like
MxR
RSV-199
MPE8
ADI-19425
LOR75
LOR87
LOR97
LOR69
LOR72
LOR19
LOR24
LOR73
LOR82
SC-DM
K42E
G307R
D310A
P312A
D385A
Binding
100%
75%
50%
25%
0%
O
HRSV-specific antibodies
Goodwin et al. (2018) 5
Gilman et al. (2016) 6
Abu-Shmais et al. (2024) 7
Count
IGHV gene
P
Generation probability (OLGA)
LOR24-like
LOR69-like
MPE8-like
Q
R
S
T
N99$_{HC}$R
S31$_{HC}$Y
S53$_{HC}$T
T73$_{HC}$R + S74$_{HC}$R

Figure S6. Evolution of the LOR69 lineage and structural basis for binding of site III mAbs. Related to Figures 5 and 6.

(A) Network map of LOR69:HRSV preF and MPE8:HRSV preF interactions as determined by the Epitope Analyzer tool.[1]

(B) Amino acid sequence alignment of mature LOR69 heavy and light chains with LOR69_UCA set as the reference. Boxes indicate the CDRs according to IMGT (gray) and Kabat (pink).

(C) Maximum-likelihood phylogenetic tree of LOR69 lineage development, rooted on the LOR69_UCA.

(D) Binding of recombinantly expressed LOR69 germline reversions by ELISA assayed against HRSV preF, HRSV postF and HMPV preF. Binding is visualized as a heatmap of -log(EC50) values derived from fitted binding curves.

(E) HRSV and HMPV neutralization log(IC50) values for recombinantly expressed LOR69 germline reversions.

(F) Magnified view of $LOR69_{LC}$ interaction with key epitope residues $I309_{F}$, $P312_{F}$ and $D344_{F}$.

(G) Magnified view of LOR69 $Y94_{LC}$ π-stacking interaction with $Y100a_{HC}$ and hydrogen bond with $K42_{F}$.

(H) Magnified view of LOR69 HCDR3 interactions with HRSV-F alpha8 helix.

(I) Magnified view of LOR69 HCDR1 and HCDR2 potentially clashing with HMPV-F alpha8 helix.

(J) Magnified view of LOR69 HFR3 interaction with HRSV-F β22.

(K) LOR69 epitope across HRSV preF and postF showing the major structural rearrangement of β22.

(L) Magnified view of $LOR69_{HC}$ interaction with $D385_{F}$.

(M) Magnified views of MPE8, LOR24 and LOR69 interactions with wild-type HRSV-F and $G307R_{F}$, $P312A_{F}$ and $K42E_{F}$ mutants, respectively.

(N) Binding of site III-targeting mAbs to HRSV-F and distinct single-point mutants. Binding is visualized as a heatmap of AUC values relative to those of reference antibodies D25 and ADI-18992.

(O) IGHV gene usage in HRSV-specific antibody repertoires following infection reported in previous studies.[5–7]

(P) Theoretical generation probabilities (Pgen) calculated from the mature antibodies belonging to each mAb class using the OLGA[8] tool.

(Q and R) Magnified views of LOR24 substitutions $S31_{HC}Y$ (Q) and $S53_{HC}T$ (R) mimicking MPE8 interactions by $Y32_{HC}$ and $T28_{HC}$.

(S and T) Magnified views of LOR24 substitutions $N99_{HC}R$ (S), and $T73_{HC}R$ and $S74_{HC}R$ (T), which may form electrostatic interactions with $D331_{F}$ and $E345_{F}$, respectively, to increase reactivity to HMPV-F.

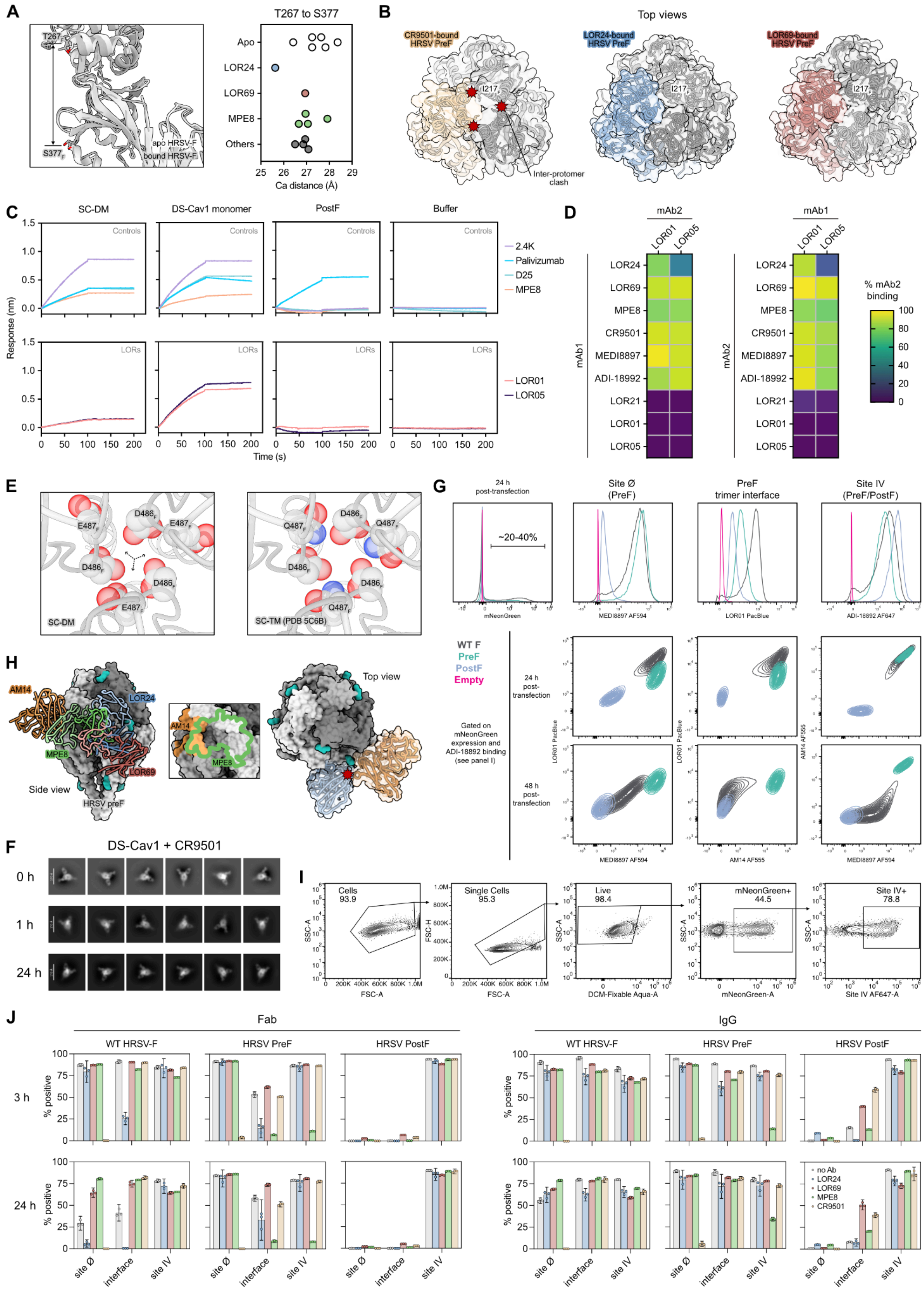

A
T267 to S377
Apo
LOR24
LOR69
MPE8
Others
Ca distance (Å)
apo HRSV-F
bound HRSV-F
B
Top views
CR9501-bound HRSV PreF
LOR24-bound HRSV PreF
LOR69-bound HRSV PreF
Inter-protomer clash
C
SC-DM
DS-Cav1 monomer
PostF
Buffer
Controls
LORs
Response (nm)
Time (s)
2.4K
Palivizumab
D25
MPE8
LOR01
LOR05
D
mAb2
mAb1
% mAb2 binding
E
SC-DM
SC-TM (PDB 5C6B)
F
DS-Cav1 + CR9501
0 h
1 h
24 h
G
24 h post-transfection
Site Ø (PreF)
PreF trimer interface
Site IV (PreF/PostF)
~20-40%
WT F
PreF
PostF
Empty
Gated on mNeonGreen expression and ADI-18892 binding (see panel I)
48 h post-transfection
H
AM14
LOR24
MPE8
LOR69
Side view
HRSV preF
Top view
I
Cells 93.9
Single Cells 95.3
Live 98.4
mNeonGreen+ 44.5
Site IV+ 78.8
J
Fab
IgG
WT HRSV-F
HRSV PreF
HRSV PostF
3 h
24 h
% positive
site Ø
interface
site IV
no Ab
LOR24
LOR69
MPE8
CR9501

Figure S7. Site III mAbs neutralize pneumoviruses through distinct mechanisms. Related to Figure 7.

(A) Compression of the LOR24 epitope (Cα distance between the peripheral residues T267 and S377) in antibody-bound HRSV preF complex compared with the apo structure (PDB 5C6B).

(B) Top views of the CR9501, LOR24 and LOR69-bound HRSV-F trimer apex. Comparison of antibody-mediated destabilization events.

(C) BLI validation of LOR01 and LOR05 preference for DS-Cav1 monomer. Responses are background subtracted.

(D) Antibody competition assays by BLI using DS-Cav1 monomer.

(E) Magnified view of the HRSV-F core in the prefusion-stabilized SC-DM and SC-TM constructs. $E487Q_F$ mutation in SC-TM abolishes the electrostatic repulsion in the SC-DM core.

(F) nsEM 2D class averages of CR9501 Fab binding DS-Cav1 + DTT over time.

(G) Characterization of HRSV-F transmembrane constructs by flow cytometry, validated using a panel of fluorescently-labeled mAbs.

(H) Side map of AM14, MPE8, LOR24 and LOR69-bound HRSV preF overlay (left). Overlap of the AM14 and MPE8 epitopes (center). Top view of the predicted clash between LOR24 and AM14 (right).

(I) Flow cytometry gating strategy for distinct conformations of the HRSV-F transmembrane constructs.

(J) Pre-gated data of the HRSV-F transmembrane assay upon addition of Fabs and IgGs at 3 h and 24 h.

Table S1. CryoEM data collection statistics.

	LOR24 PostF	LOR24 SC-DM	LOR69 SC-DM
**Access codes**			
PDB	PDB_000038DV	PDB_000038GD	PDB_000033SD
EMDB	EMD-78752	EMD-78790	EMD-59533
**Data collection & processing**			
Microscope	Glacios	Titan Krios	Glacios
Voltage (kV)	200	300	200
Detector	Gatan K3	Gatan K3	Falcon IV
Recording mode	Counting	Counting	Counting (EER)
Magnification	45,000 X	105,000 X	150,000 X
Movie micrograph pixel size (Å)	0.885	0.84	0.926
Dose rate ($e^-/Å^2/s$)	10	14.172335	8.56
No. of frames per movie micrograph	100	100	
Frame exposure time (ms)	50	40	
Number of EER fractions			40
Movie micrograph exposure time (s)	5	4	4.67
Total dose ($e^-/Å^2$)	50	56.68934	40
Under focus range (µm)	0.8-1.8	0.8 - 1.5	-0.5 - -0.25
Number of movie micrographs	2,190	11,678	5,031
**Model refinement and validation**			
Map resolution (A)	3.67	3.09	3.3
Map symmetry	C3	C3	C3
Non-Hydrogen Atoms	12848	14253	15687
Residues	1824	1938	2016
Carbohydrates	3 NAG	9	9
RMSD Bonds	0.004	0.004	0.020
RMSD Angles	0.740	0.559	1.622
**Ramachandran**			
Outliers (%)	0.06	0.16	0.00
Allowed (%)	3.80	4.32	3.31
Favored (%)	96.14	95.52	96.69
Rotamer outliers	0.06	1.02	0.00
Clash score	4.02	5.88	3.57
Molprobity score	1.42	1.64	1.36
EMRinger score	2.75	2.60	2.4

Supplementary References

1. Montiel-Garcia, D., Rojas-Labra, O., Santoyo-Rivera, N., and Reddy, V.S. (2022). Epitope-Analyzer: A structure-based webtool to analyze broadly neutralizing epitopes. J. Struct. Biol. *214*, 107839. https://doi.org/10.1016/j.jsb.2022.107839.

2. Abramson, J., Adler, J., Dunger, J., Evans, R., Green, T., Pritzel, A., Ronneberger, O., Willmore, L., Ballard, A.J., Bambrick, J., et al. (2024). Accurate structure prediction of biomolecular interactions with AlphaFold 3. Nature *630*, 493–500. https://doi.org/10.1038/s41586-024-07487-w.

3. Ols, S., Lenart, K., Cerveira, R.A., Miranda, M.C., Brunette, N., Kochmann, J., Corcoran, M., Skotheim, R., Philomin, A., Cagigi, A., et al. (2023). Multivalent antigen display on nanoparticle immunogens increases B cell clonotype diversity and neutralization breadth to pneumoviruses. Immunity *56*, 2425-2441.e14. https://doi.org/10.1016/j.immuni.2023.08.011.

4. Briney, B., Inderbitzin, A., Joyce, C., and Burton, D.R. (2019). Commonality despite exceptional diversity in the baseline human antibody repertoire. Nature *566*, 393–397. https://doi.org/10.1038/s41586-019-0879-y.

5. Goodwin, E., Gilman, M.S.A., Wrapp, D., Chen, M., Ngwuta, J.O., Moin, S.M., Bai, P., Sivasubramanian, A., Connor, R.I., Wright, P.F., et al. (2018). Infants Infected with Respiratory Syncytial Virus Generate Potent Neutralizing Antibodies that Lack Somatic Hypermutation. Immunity *48*, 339-349.e5. https://doi.org/10.1016/j.immuni.2018.01.005.

6. Gilman, M.S.A., Castellanos, C.A., Chen, M., Ngwuta, J.O., Goodwin, E., Moin, S.M., Mas, V., Melero, J.A., Wright, P.F., Graham, B.S., et al. (2016). Rapid profiling of RSV antibody repertoires from the memory B cells of naturally infected adult donors. Sci. Immunol. 1, eaaj1879. https://doi.org/10.1126/sciimmunol.aaj1879.

7. Abu-Shmais, A.A., Vukovich, M.J., Wasdin, P.T., Suresh, Y.P., Marinov, T.M., Rush, S.A., Gillespie, R.A., Sankhala, R.S., Choe, M., Joyce, M.G., et al. (2024). Antibody sequence determinants of viral antigen specificity. mBio *15*, e01560-24. https://doi.org/10.1128/mbio.01560-24.

8. Sethna, Z., Elhanati, Y., Callan, C.G., Walczak, A.M., and Mora, T. (2019). OLGA: fast computation of generation probabilities of B- and T-cell receptor amino acid sequences and motifs. Bioinformatics *35*, 2974–2981. https://doi.org/10.1093/bioinformatics/btz035.
